# Laparoscopic Nephrectomy versus Open Nephrectomy for Patients with Autosomal Dominant Polycystic Kidney Disease: A Systematic Review and Meta-Analysis

**DOI:** 10.1371/journal.pone.0129317

**Published:** 2015-06-08

**Authors:** Pengyu Guo, Wanhai Xu, Huibo Li, Tong Ren, Shaobin Ni, Minghua Ren

**Affiliations:** 1 Department of Urinary Surgery, the First Affiliated Hospital of Harbin Medical University, Harbin, Heilongjiang Province, China; 2 Department of Urinary Surgery, the Fourth Affiliated Hospital of Harbin Medical University, Harbin, Heilongjiang Province, China; 3 Department of Gastroenterology, the Second Affiliated Hospital of Harbin Medical University, Harbin, Heilongjiang Province, China; University of Florida, UNITED STATES

## Abstract

**Objective:**

To compare efficacy and safety of laparoscopicnephrectomy (LN) versusopen nephrectomy (ON) in the management of autosomal dominant polycystic kidney disease (ADPKD), we conducted a systematic review and meta-analysis.

**Methods:**

A systematic search of the electronic databases PubMed, Scopus, and the Cochrane Library was performed up to October 2014.This systematic review was performed based on observational comparative studies that assessed the two techniques. The weighted mean difference (WMD) and risk ratio (RR), with their corresponding 95% confidence interval (CI), were calculated to compare continuous and dichotomous variables, respectively.

**Results:**

Seven studies were identified, including 195 cases (118 LN / 77 ON). Although LN was associated with longer operative time (WMD 30.236, 95%CI 14.541 −45.932, P<0.001) and the specimen might not have been resected as heavy as the ON group (WMD -986.516, 95%CI -1883.24–-89.795, P = 0.031), patients in this group might benefit from a shorter length of hospital stay (WMD -3.576, 95%CI 4.976–-2.176, P <0.001), less estimated blood loss (WMD -180.245, 95%CI -317.939–-42.556, P = 0.010), and lower need of transfusion (RR 0.345, 95%CI 0.183–0.650, P = 0.001). The LN group also had less overall complications (RR 0.545, 95%CI 0.329–0.903, P = 0.018). The need of narcotic analgesics between the two groups might have no significant difference (WMD -54.66, 95%CI -129.76–20.44, P = 0.154).

**Conclusion:**

LN for giant symptomatic ADPKD was feasible, safe and efficacious. Morbidity was significantly reduced compared with the open approach. For an experienced laparoscopist, LN might be a better alternative.

## Introduction

Autosomal dominant polycystic kidney disease (ADPKD), a genetically heterogeneous disease in which 2 genes are involved, PKD1 (chromosome16p13.3) and PKD2 (chromosome 4q21-23)[1], is the fourth commonest cause (approximate 10%) of all cases of end stage renal disease[2].Approximate 50% of ADPKD patients ultimately require dialysis or renal transplantation by the age of 60[3], with a loss of 4.4–5.9 ml/min in the GFR[4]. Patients with ADPKD have several other complications, such as cysts in the liver, spleenand lungs[5], and aneurysms of the circle of Willis (Berry aneurysms), which lead to death in 8% to 11% of patients with ADPKD[6]. Massively enlarged nonfunctionalpolycystic kidneys may contribute to abdominal pain, hematuria, hemorrhage, hypertension and infection. The purpose of the current treatment approach is to relieve pain and limit morbidity and mortality. Nephrectomy (unilateral or bilateral) is not required for most patients.Only 20% of patients with ADPKD require nephrectomy[7]. However, in patients with end-stage renal disease who have a history of infected cysts and frequent hemorrhaging, it is generally accepted that nephrectomy can be undertaken for symptomatic relief of pain [8]. Traditionally, nephrectomy before transplantation has been accepted[9], although the timing and indication for nephrectomy in patients with ADPKD arestill controversial.

Open surgery needs a large subcostal incision andcan cause significant complication.[10] Laparoscopicnephrectomy (LN) now is a mature and applicable surgical approach for some renal conditions, resulting in better short-term outcomes.[11,12]. Compared withthe normal-size kidney, LN for ADPKD is challenging due to the massive size of these kidneys[13] and there is a difficulty in negotiating this large mass with the laparoscopic approach[14]. A large incision for specimen extraction after LN maycompromise the final cosmetic result of the laparoscopic procedure[15]. In the meanwhile, incision-related complications may occur. Therefore, unlike LN on most of renal conditions, whether LN for ADPKD is a better alternative is controversial. Since Elashryet al[16] first reported LN for ADPKD in 1996, it has been adopted in many large medical centers, demonstrating its feasibility[17–19]. Recently, several studies comparing LN with ON for ADPKD were reported but with controversial results.[14,20–24]We pooled the data of available literature to perform a systematic review using meta-analysis to compare LN with ON for patients with ADPKD and determine whether LN is a better option.

## Materials and Methods

There was no review protocol for our study, and no registration information can be provided for this systematic review.

### Search Strategy

Asystematic literature search, restricted to the English language articles, was performed in electronic databases, including PubMedand the Cochrane Library, up to October 2014. The following terms were used: “laparoscopic and polycystic and nephrectomy”. The Related Articles function was also appliedto broaden the search. We used the most recent or complete report, when multiple reports described the same population.

### Inclusion Criteria

Studies included in our study had to meet all of the following criteria: (a) randomized controlled trials (RCTs) or observational comparative studies comparing LN with ON,(b) patients must be diagnosed with ADPKD, (c) at least one outcome of interests was included. (d) data from selected studies couldbe used for meta-analysis directly or could be calculated using statistical formula[25]. Review articles, experimental animal studies and incomplete documentations such as comments, conference abstracts were excluded. Studies of which patients’ characteristics were obviously different from regular ones, after evaluated by Metaninf function in. ata 12.0, were excluded in the final analysis as well.

### Data Extraction

Two separateinvestigators independently screened the eligible studies and extracted data from them.Disagreements were resolved by discussion.Data extracted from eligible studies included the first author, publication year, country, study design, matching criteria and the outcomes of interests. Since the data reported by Mary Eng et al[20]wasdivided into two subgroups, Mary Eng et al(unilateral) and Mary Eng et al (bilateral), we considered the datatwo studies when conducting the meta-analysis.

### Statistical analysis

The meta-analysis was performed using Stata 12.0.The weighted mean difference (WMD) and the risk ratio (RR) were used to calculate continuous variables and dichotomous variables respectively.When continuous variables were measured in different units, we used the standardized mean difference (SMD) to minimize the effects of heterogeneity. Means and standard deviations (SD) were calculated using statistical formulain those studies that provided continuous data as median and range values.Statistical heterogeneity among all studies was evaluated by the chi-squared test with significance set at P < 0.10, and the quantity ofheterogeneity was evaluated using the I^2^ statistic. A random-effects model was used for outcomes that showed significant heterogeneity between studies (I^2^>50). Otherwise, we used the fixed-effects model[26]. This meta-analysis wasin accordance with the guidelines of the Preferred Reporting Items for Systematic Reviews and Meta-analyses (PRISMA) statement.[27]

Subgroup analysis was performed. Since the primary observed complications were mainly wound complications and hand-assisted laparoscopic nephrectomy (HALN) needed surgical incision for hand, studies reporting overall complications were dividedinto two subgroups according to HALN and pure LN. Sensitivity analysis was performed using the Metaninf function in Stata 12.0.Publication bias was not assessed because the Cochrane Collaboration guidelines didnot recommend testing asymmetry of funnel plots in a meta-analysis within 10 trials, which was the case in our meta-analysis.[28]

According to the criteriaprovided by the Centre for Evidence-Based Medicine in Oxford, UK, we rated studies for the level of evidence[29], and evaluatedmethodological quality of observational comparative studiesby the modified Newcastle-Ottawa scale.[30] A score of 0–9 was used toassessed each study.

## Results

**Fig 1:**
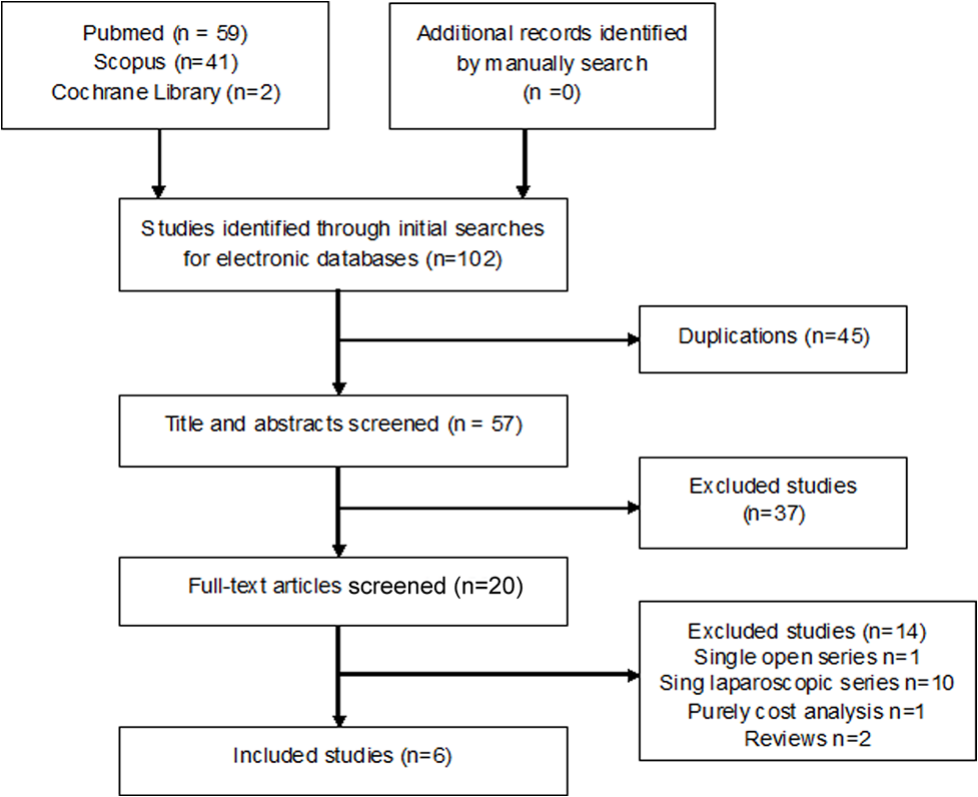
PRISMA flow chart of studies identified, included, and excluded in meta-analysis.

Six published articles that contained seven studies[14,20–24], including 195 cases (118 cases for LN and 77 cases for ON), were included in the final analysis (**Fig 1**). Characteristics of the included studieswere shown in **Table 1**. The quality of the included studies was not high, and no RCTs was included. One literature wasprospectivestudy[24] and other five were retrospective studies[14,20–23]. None of the retrospective studies declared proper treatment allocation. One of them had an evidence level of 4[21], the other five had evidence levels of 3. Of all comparative studies, only one[21]had a score of 5, the other five had a score of ≥6. Seven outcomes were used to compare LN and ON. Perioperative variables: operating time (min), estimated blood loss (EBL, ml), specimen weight (g) and transfusion (%), Postoperative variables: length of hospital stay (LOS, day), overall complications and analgesic requirement (morphine equivalents, mg). **Table 2**showedoverall analysis between the two techniques.

**Table 1:** Characteristics of included studies.

Studies	Year	Country	Study type	No.patients LN/ON	BMI LN/ON	ASA	Level of evidence*	Matching factors	Quality scores^#^	Variables
Seshadri	2001	Canada	Prospective	10/10	NA	N	3	1,2,5,6,7,8	6	1,2,4
Gill	2001	USA	Retrospective	10/10	35.9/23.8	3	3	1,2,3,4,7,8	6	1,2,4,5,6
Binsaleh	2006	Canada	Retrospective	6/6	29/26	3	3	1,2,3,4,5	6	1,2,3,4,5
Desai	2007	India	Retrospective	13/14	26.3	N	4	1,2,3,5,7	5	1,2,3,4,6
Verhoest	2012	France	Retrospective	21/19	25/23	3	3	1,2,3,6,7,8	6	1,2,4,5,6,7
Eng(Unilateral)	2013	USA	Retrospective	24/12	NA	N	3	1,2,3,5,6,7,8	7	1,2,3,4,7
Eng(Bilateral)	2013	USA	Retrospective	34/6	NA	N	3	1,2,3,5,6,7,8	7	1,2,3,4,7

Matching factors:1,age; 2,gender; 3,body mass index; 4,American Society of Anesthesiology score; 5,operative side; 6,preoperative dialysis; 7,Indication for surgery; 8, kidney size; 9, previous pelvic/abdominal surgery; Variables:1,operative time; 2,length of hospital stay; 3, specimen weight; 4,overall complications; 5,estimated blood loss; 6, Analgesic requirement; 7, Transfusion. BMI: Body Mass Index, NA: data not available.

*Level of evidence: according to criteria by the Centre for Evidence-Based Medicine[28].

^#^ Quality scores: according to criteria by the Newcastle-Ottawa Scale[29].

**Table 2:** Overall analysis of LN vs. ON.

			Statistical results			Study heterogeneity			
Outcome	No. of studies	LN/ON	Statistic	Value(95%CI)	P	χ^2^	df	Iflue	P
Operative time(min)	7	118/77	WMD	30.23(14.54,45.93)	<0.001	7.78	6	23.8	0.248
LOS(day)	7	118/77	WMD	-3.576(-4.976,-2.176)	<0.001	19.92	6	69.9	0.003
Specimen(g)	5	87/48	WMD	-986.5(-1883,-89.80)	0.031	22.64	4	82.3	<0.001
Analgesic(mg)	3	37/35	WMD	-54.66(-129.8,-20.44)	0.154	6.47	2	69.1	0.039
EBL(ml)	3	37/35	WMD	-180.3(-317.9,-42.57)	0.01	4.2	2	52.4	0.122
Complication(%)	5	95/53	OR	0.545(0.329,0.903)	0.018	1.14	4	0	0.888
Transfusion(%)	3	79/37	OR	0.345(0.183,0.650)	0.001	0.14	2	0	0.932

Overall analysis between the two techniques.

### Operative time

**Fig 2:**
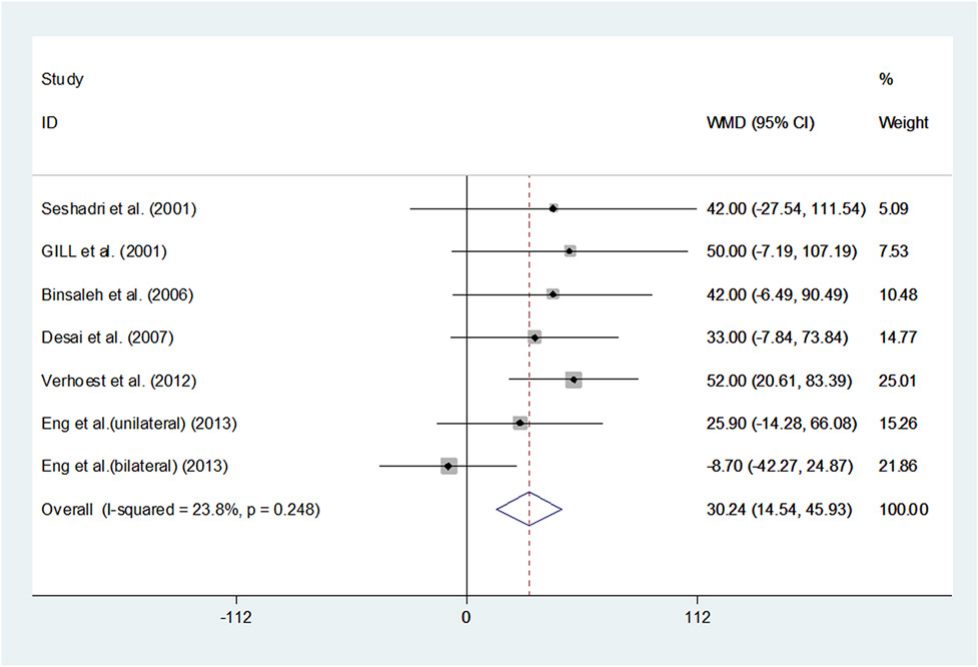
Forest plots of operative time using a fixed-effect model. Squares indicate study-specific risk estimates (size of the square reflects the study-specific statistical weight, i.e., the inverse of the variance); horizontal lines indicate 95% confidence intervals (CIs); diamonds indicate summary risk estimate with its corresponding 95% confidence interval.

Pooling the data of the seven studies[14,20–24] including 195 patients, demonstrated significantly longer operative time in the LN than the ON group (WMD 30.236, 95%CI 14.541–45.932, P<0.001),suggesting thatthe necessary operative time might be longer in the LN group. (**Fig 2**) When calculating the operative time, we only found Mary Eng et alshowed no significant differences between the two groups.

### Estimated blood loss

**Fig 3:**
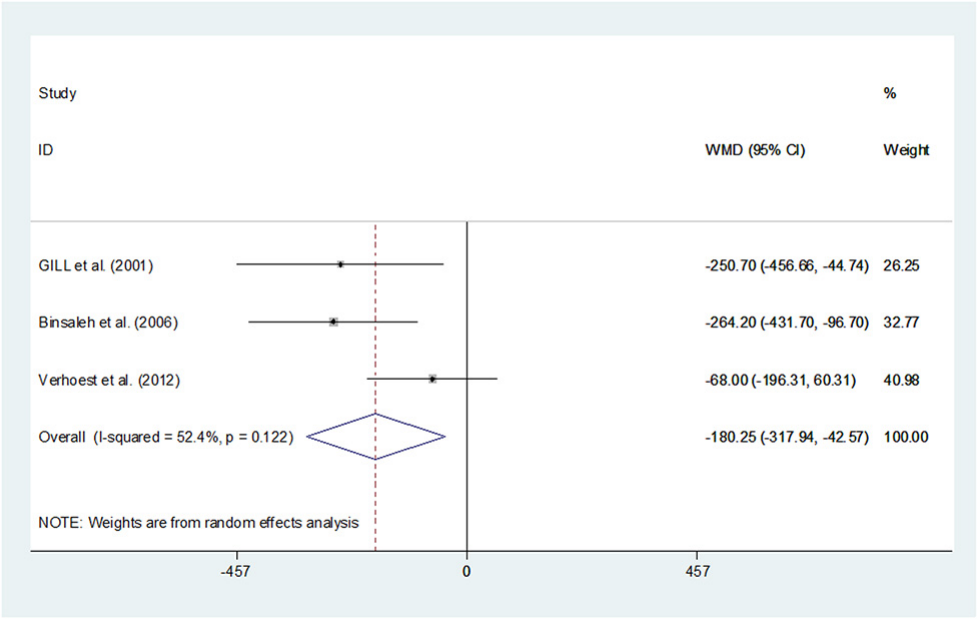
Forest plots of estimated blood loss using a random-effect model. Squares indicate study-specific risk estimates (size of the square reflects the study-specific statistical weight, i.e., the inverse of the variance); horizontal lines indicate 95% confidence intervals (CIs); diamonds indicate summary risk estimate with its corresponding 95% confidence interval.

Pooling the data from the three studies[14,22,23] showed a significant difference between LN and ON regarding to EBL (WMD -180.245, 95%CI -317.939–-42.556, P = 0.010). The LN group might have less EBL than the ON group. (**Fig 3**)

### Specimen weight

**Fig 4:**
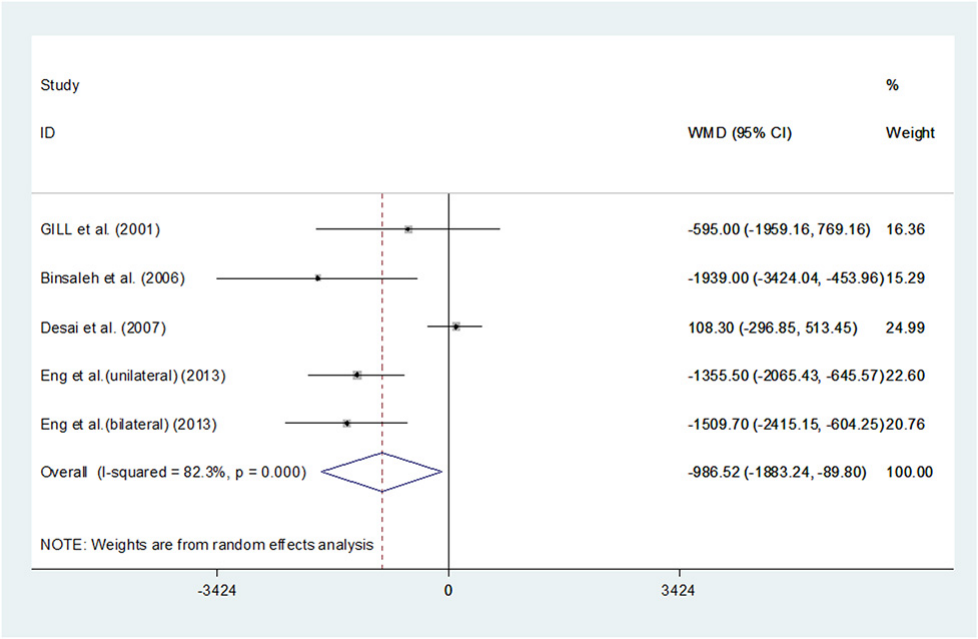
Forest plots of specimen weight using a random-effect model. Squares indicate study-specific risk estimates (size of the square reflects the study-specific statistical weight, i.e., the inverse of the variance); horizontal lines indicate 95% confidence intervals (CIs); diamonds indicate summary risk estimate with its corresponding 95% confidence interval.

Pooling the data of the five studies[14,20–23]reporting specimen weight significantly showed that LN might not extract specimens that wereas heavy as the ON group (WMD -986.516, 95%CI -1883.24 –-89.795, P = 0.031). However, it hadbeen reported that gross specimenscouldbe extracted through the laparoscopic approach[22]. The results suggested that ON might be safer and more feasible for patients with largerspecimens. (**Fig 4**) Since only one prospective study and no RCTs was included in our study, we could not get to know whether there was a pre-selection prior to the surgeon's decision on LN or ON, and this may influence the evidence level of the results we calculated.

### Transfusion

**Fig 5:**
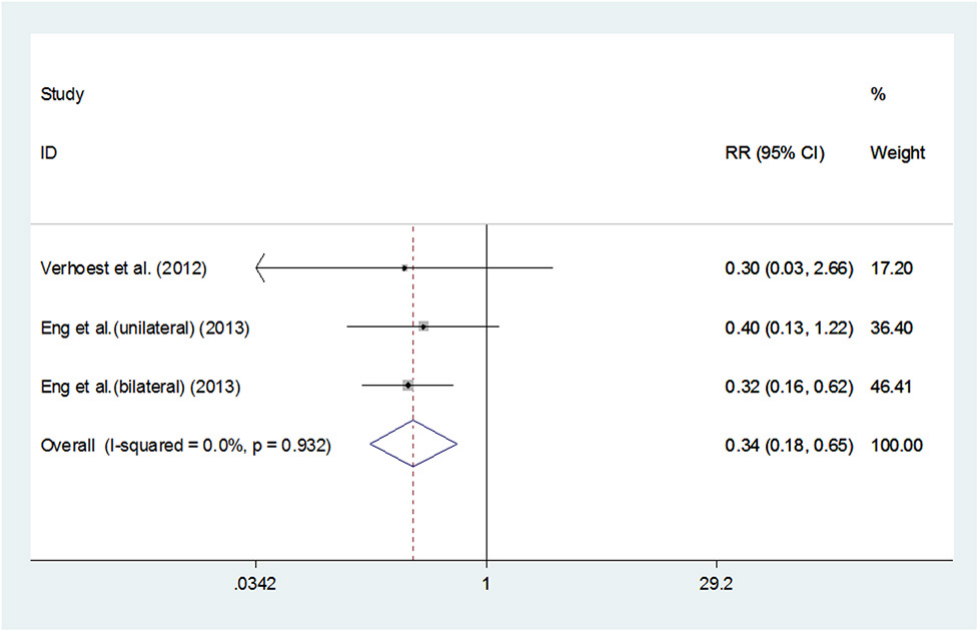
Forest plots of transfusion using a fixed-effect model. Squares indicate study-specific risk estimates (size of the square reflects the study-specific statistical weight, i.e., the inverse of the variance); horizontal lines indicate 95% confidence intervals (CIs); diamonds indicate summary risk estimate with its corresponding 95% confidence interval.

Pooling data of the three studies[20,23]that reported transfusion showed a significant difference, the LN group had a reduced need of transfusion(RR 0.345, 95%CI 0.183–0.650, P = 0.001). Reduced need of transfusion was associated with less EBL, which had been proven above. (**Fig 5**)

### LOS

**Fig 6:**
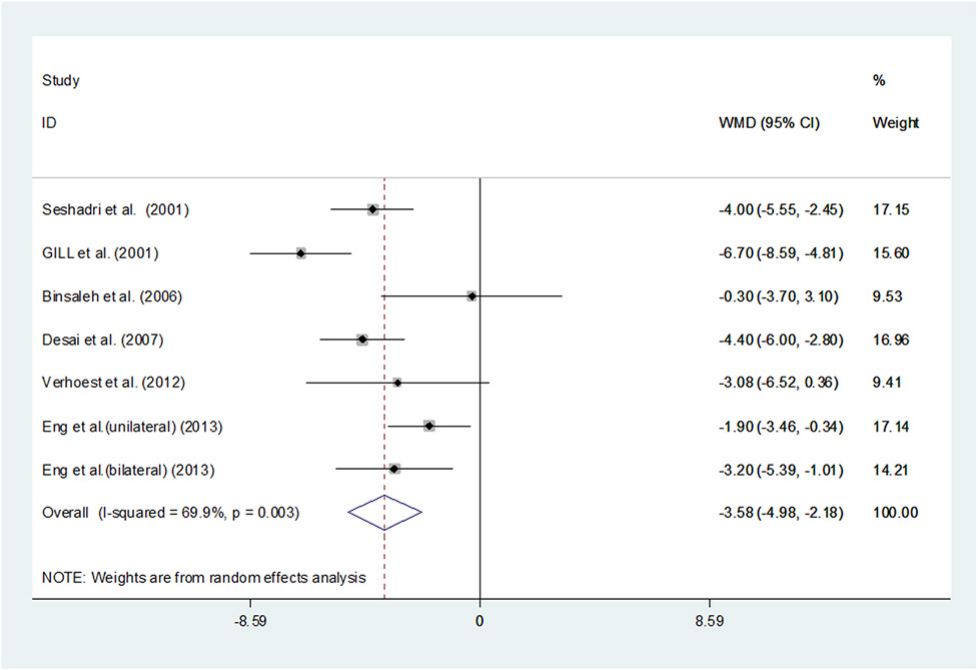
Forest plots of length of hospital stay using a random-effect model. Squares indicate study-specific risk estimates (size of the square reflects the study-specific statistical weight, i.e., the inverse of the variance); horizontal lines indicate 95% confidence intervals (CIs); diamonds indicate summary risk estimate with its corresponding 95% confidence interval.

Pooling data of the seven studies[14,20–24]including 195 patients showed a significantly shorter LOS in the LN group than the ON group (WMD -3.576, 95%CI -4.976 –-2.176, P <0.001). (**Fig 6**) All the studies showed LN had shorter LOS than ON, which met our expectations.

### 3.6 Overall complications

**Fig 7:**
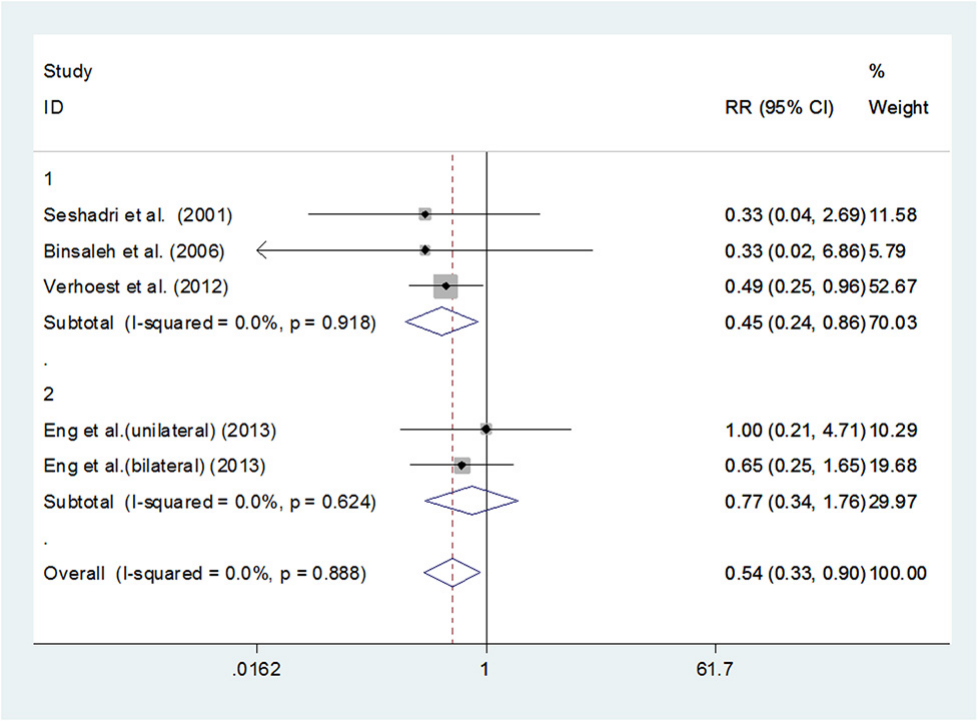
Forest plots of overall complicationsusing a fixed-effect model. Studies are sub-grouped according to pure laparoscopic nephrectomy and hand-assisted laparoscopic nephrectomy. Squares indicate study-specific risk estimates (size of the square reflects the study-specific statistical weight, i.e., the inverse of the variance); horizontal lines indicate 95% confidence intervals (CIs); diamonds indicate summary risk estimate with its corresponding 95% confidence interval.

Pooling data of the five studies[2,14,23,24]that reported complications showed that the LN group had significantly fewer complications than the ON group (RR 0.545, 95%CI 0.329–0.903, P = 0.018); however, subgroup study suggested that there wasno significant difference between HALN and ON (RR 0.768, 95%CI 0.335–1.760, P = 0.533). **(Fig 7**)

### Analgesic requirement

**Fig 8:**
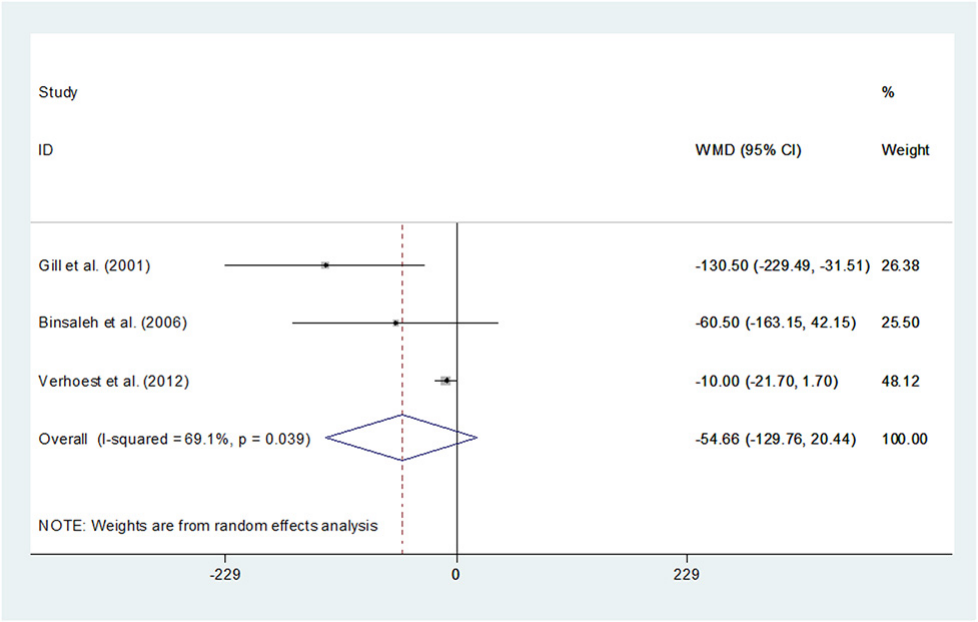
Forest plots of analgesic requirement using a random-effect model. Squares indicate study-specific risk estimates (size of the square reflects the study-specific statistical weight, i.e., the inverse of the variance); horizontal lines indicate 95% confidence intervals (CIs); diamonds indicate summary risk estimate with its corresponding 95% confidence interval.

The measurement of analgesic wasconverted into morphine(mg). Pooling data of the three studies[14,22,23]that reported analgesic requirement showed there wasno statistical significance between the two groups (WMD -54.66 95%CI -129.76–20.44, P = 0.154). (**Fig 8**) However, three studies reported that analgesic requirement in the LN group waslower than the ON group, respectively. As the sample size was small and the random-effect model was used due to significant heterogeneity, we should be cautious in the interpretation of the results as further study would beneeded to substantiate and update this conclusion.

### Sensitivity analysis

**Fig 9:**
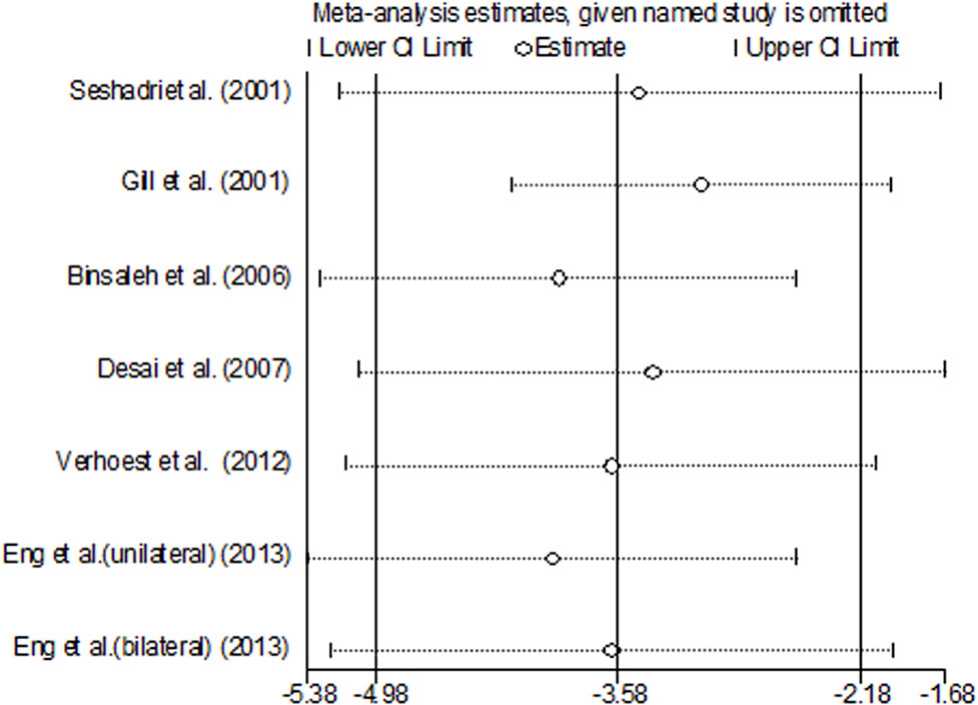
Sensitive analysis of all the studies using Metaninf function in Stata 12.0.

We pooled the data of seven studies using the Metaninf function in Stata 12.0. This function estimated the influence of each individual study on the overall meta-analysis summary estimate. The command presented a graph of the results of an influence analysis in which the meta-analysis was re-estimated by omitting each study in turn. The figure of every parameter showed that all of the estimated points were in the CI of the final results, which suggestedthat none of the included studies were obviously different from the others and the results we calculated werereliable. We showed the figure of LOS, because this parameter contained all of the included studies, and the result of the data statistics was representative of the overall sensitivity analysis. **(Fig 9)**

## Discussion

The indication of nephrectomy for patients with ADPKD contained renal, extrarenal complications, organ compression or the need of making space for the renal transplant prior to transplantation.[31]It hadbeen reported that the overall risk of performing a nephrectomy waslower than the potential complications, including graft loss[32]. Open nephrectomy (ON) wasassociated with high mortality and morbidity.[33]Pooling data indicatedthat LN might have longer operative time, shorter LOS, a lighterextracted specimen, lower overall complication rate, less EBL and lower transfusion rate;however, the analgesic requirement had no significant difference between LN and ON.

A LN was less likely to cause blood loss than an ON, mostly due to the creation of pneumoperitoneum[20].Lower EBL mightcontribute to decreased perioperative mortality and morbidity.[34] For patients who considered renal transplantation, it wasbetter not to receive a blood transfusion before transplantation because production of antihuman leukocyte antigen antibodies mightoccur after transfusion.[35–37] The sensitized patientsmighthave difficulties in locating compatible organs for transfusion. These factors werethe primary advantages of LN that should be appreciated. Pooling the data of the analgesic requirement variable showed that LN and ON had no significant difference; however, all the three studies reported that patientsof LN group had a reduced need ofanalgesics than those of ON. Because the I^2^ value of this meta-analysis was69.1% (>50%) and a random-effect model was used, the results should be interpreted with caution. As there were differences in postoperative patient management and criteria, it was unknown whether the potential benefit couldbe proven in future well-designed RCTs.

Complication wasone of the most frequent-considered and important points to evaluate surgical procedure[38]. In 1973, Bennett et al[10] reported 31 patients who underwent bilateral open nephrectomy for ADPKD with a morbidity rate of 38% and a mortality rate of 3%. Since then, many improvements in operative techniques, including minimally invasive techniques like LN, occurred, which significantly contributed tobetter perioperative parameters. As intraoperative complication rate and postoperative complication rate were not available in every study, we only calculated the overall complication rate for meta-analysis. In this review, LN had a lower perioperative complication rate than the LN group, which indicated that, compared with ON,LN might be safer and more effective. Although LN requireda pneumoperitoneum and might cause some adverse effects, such as pulmonary disorders and subcutaneous emphysema, less pulmonary complications were found in the LN group than that in the ON group.[20]It waspossible that reduced pain was the reason whyearlier pulmonary rehabilitation could be promoted. In addition, EBL was less in the LN group, which might reduce complications as well. Subgroup analysis was performed and studies reporting overall complications were dividedinto two subgroups according to the procedure, hand-assisted laparoscopic nephrectomy (HALN) and pure LN. The primary observed complications were mainly wound complications. The surgicalincision for hand mightbe the reason why the complication rate between HALN and ON had no significant difference.

The operative time of laparoscopic approach was significant longer. The longer time mightbeinsignificant due to today’s limited resources. Gill et al[22] reported that, in their study, although the laparoscopic approach took an average of 36 minutes longer than the open approach, the operative time ofLN included 45 minutes for patient repositioning, which wasnot necessary for ON. We werenot sure whether other studies hadused the same procedure, and whether it had been taken into account. Some studies also indicated that the operative time woulddecrease with increasing experience in the use of the laparoscopic procedure. Eng et al[20] reported that, as with other procedures, there was a learning curve. When they compared the operative time ofthose who performed <5 or >10 procedures, they observed a trend ofa longer operative time for the less experienced surgeons. We also found that, compared with pure LN, HALN had shorter operative time but higher complication rate. Nevertheless, further study would beneeded for verification, especially verificationat a multi-institutional level.

Unlike the normal-sized kidney, LN for ADPKD wouldbe challenging due to the massive size of these kidneys. The results of our study showed that the specimen size (by weight) was greater in the open group. Desai et al[39] indicated that through the larger midline incision used in the open technique, the massive kidney could often be removed intactly with minimal rupture of cysts. While in the LN group, cysts were deliberately ruptured to extract the enlarged kidneys through the smaller incision. This resulted in a lighterweight of the specimen but didnot necessarily mean that size of the kidneys was smaller. Some authors advocated reducing the kidney volume intracorporeally by cyst puncture[18]; however, some urologists found it hazardous as it could cause peritonitis-like symptoms or prolonged ileus[40] because most of the cyst fluid wasinfectious. As the laparoscopic nephrectomy couldbe performed viaa transperitoneal orretroperitoneal approach, the retroperitoneal approach mightbe a better option to avoid the symptoms above.[41] Nevertheless, foran increased kidney size, the transperitoneal route was more convenient because of the larger space it offered. In the retroperitoneal approach, Gill et al[22] demonstrated that secondary balloon dilation of the upper and lower retroperitoneum external to Gerota’s fascia was performed routinely to create a larger working space. This procedure wasdifferent from the transperitoneal approach. The largest specimen weight of one kidney in their series was 2.6 kg, which was a considerab heavy kidney that could beretrieved laparoscopically. Considering its longer operative time, limitation oflighter specimen and potential risks such as injury to the intra-abdominal organs due to limited abdominal working space, some authors advocated that hand-assisted laparoscopic nephrectomy shouldbe more feasible. It wasmore useful for a bilateral nephrectomy in order to minimize the operative time and the technical challenge was associated with a pure laparoscopic nephrectomy.[40] The use of LN, HALN or ON, and transperitoneal or retroperitoneal approach should be balanced in many ways.

Cost-effectiveness analysis was not performed in this meta-analysis. Yamataka et al[42] concluded that: LN was recommended for its efficiency and satisfaction with postoperative cosmesis.

ADPKD wasnot a common disease. Only patients with end-stage renal disease needing renal transplantationwere suggested to undertake nephrectomy.Thus only 6 independent studies wereincluded in our analysis, and most of the studies hadsmall sample sizes. We conducted a systematic review through meta-analysis approach. From statistical perspectives, the evidence level was not very high becausethe data strength wasweak. Currently, this wasthe first systematic review to indicate LN wasa safe and feasible approach for patients with ADPKD, or a better option if it would be conducted by experienced surgeon. Our study could be a reference for clinical practice.

The present meta-analysis has some limitations that must be taken into account. The main limitation wasthat there were few studies that were eligible for inclusion in our study.The lack of high quality RCTsmightimpact the quality of our study. Furthermore, the sample size of these studies was small and the studies adopted different matching criteria and measurement of outcomes. It was impossible tomatch all patient groups with differentage, BMI, and previous abdominal history.All of these factors could contribute to the high heterogeneityamong studies. Employingthe random model for pooling data might minimize the effects of heterogeneity but wouldnot eliminate them. We calculated the mean (SD) values from data ranges, and the bias of the pooling effect should be considered as well. Moreover,some authors did not report the pre-selection criteria prior to the surgeon's decision whetherLN or ON was should be used, which mightinfluence the reliability of the conclusions.

## Conclusions

In addition to the available evidence, for an experienced surgeon, laparoscopic nephrectomy wasa safe and feasible alternative comparing with the open approach in ADPKD patients requiring nephrectomy. LN for ADPKD wouldbe safely performed without increased complications. Considering the initial learning curve, further experience wouldprove the ability to manage more complex large kidneys in a similar minimally invasive technique.

## Supporting Information

S1 PRISMA Checklist (DOC)
